# Stress-Adaptive Training: An Adaptive Psychomotor Training According to Stress Measured by Grip Force

**DOI:** 10.3390/s22218368

**Published:** 2022-11-01

**Authors:** Yotam Sahar, Michael Wagner, Ariel Barel, Shraga Shoval

**Affiliations:** 1 Department of Industrial Engineering & Management, Ariel University, Ariel 4076414, Israel; 2 The Faculty of Computer Science, Technion-Israel Institute of Technology, Haifa 3200003, Israel

**Keywords:** grip force, stress, adaptive training, psychomotor training, psychomotor tasks, physiological indices

## Abstract

Current training methods show advances in simulation technologies; however, most of them fail to account for changes in the physical or mental state of the trainee. An innovative training method, adaptive to the trainee’s stress levels as measured by grip force, is described and inspected. It is compared with two standard training methods that ignore the trainee’s state, either leaving the task’s level of difficulty constant or increasing it over time. Fifty-two participants, divided into three test groups, performed a psychomotor training task. The performance level of the stress-adaptive group was higher than for both control groups, with a main effect of t = −2.12 (*p* = 0.039), while the training time was shorter than both control groups, with a main effect of t = 3.27 (*p* = 0.002). These results indicate that stress-adaptive training has the potential to improve training outcomes. Moreover, these results imply that grip force measurement has practical applications. Future studies may aid in the development of this training method and its outcomes.

## 1. Introduction

*“Natural Selection acts by the simple preservation of those individuals which are best adapted to the complex contingencies to which all are related.”*

Charles Darwin [1].

Psychomotor control is the process in which cognitive processes are used for controlling and coordinating the muscles and limbs involved in the performance of motor skills [2]. Psychomotor skills are of paramount importance in various fields such as military [3], medical [4], industrial [5], aviation [6], and more. Acquisition of psychomotor skills can be achieved by simulation training, a process through which actions are imitated and exercised in controlled environments with the intention of applying the skills to real-life scenarios [7]. However, simulator training time is still very expensive and in some cases is a rare commodity which must derive the most benefit possible with minimal use of special resources [8,9,10]. State-of-the-art innovative methods are applied to simulators in various fields in order to improve learning and training [11,12,13]. In the current research we propose an innovative training method that can improve the training process with optimal use of the resources, namely Stress-Adaptive Training (SAT).

Adaptive training is a form of training in which the task varies as a function of a certain feature or state of the trainee. Performance was the initial feature that triggered adaptability in training [14]. The notion of adaptive training is based on the assumption that a demanding task can be learnt more efficiently if it is presented throughout training at a level of complexity that is optimally matched to each individual’s current proficiency. However, performance-based adaptive training does not always capture all the individual’s relevant aspects that impact learning [14]. In a meta-analysis of 22 studies, performance-adaptive training was found to have a significant benefit of 32% on learning rate and transfer effectiveness was improved compared with fixed or increasing difficulty training methods [15].

Self-controlled task difficulty is a simple method of adjusting the training complexity to the state of the trainee. It was found to have a positive effect on training under certain conditions [15]. In this type of training, the trainee has the option to self-manage the workload related to the process during training, which increases the efficiency of learning. However, this freedom of choice yields another source of load on the trainee which has been found to overshadow the benefits of the method [16]. The need for taking into account the cognitive state of the trainee has been acknowledged in recent years [14,17]. Recently, there have been few attempts to realize workload-adaptive training systems [18,19]. However, considering workload in adaptive training may not reach its full potential. In order to adjust adaptive training to complicated tasks, it is required to investigate one of the factors affecting performance, namely the stress level of the trainee [14,20].

Stress is defined as *“… a real or interpreted threat to the physiological or psychological integrity of an individual that results in physiological and/or behavioral responses”.* [21] It has numerous effects on human capacities, including attention, memory, and performance [22]. Psychomotor performance tends to be degraded by stressful conditions, including in simulator training [23,24]. However, some findings show that under certain conditions, psychomotor performance may not be affected by stress, especially when the task is well-learned [25,26]. On the other end of the scale, extremely low stress, alternatively defined as “understimulation”, is thought to impair the ability of the person to maintain a wakeful state [27]. A concept that captures both ends of the phenomenon is the well-known inverted U, which argues that as stress levels increase, performance improves, up to a certain point, after which there is a decrease in the individual’s performance [28].

Since stress at its extremely high and extremely low levels may take a toll on performance, which affects training quality, it is of immense value to be able to recognize these situations and counteract them in real-time [20]. The measurement of stress intensity in real-time is typically performed using physiological measurements of manifestations of the sympathetic nervous system [29], such as maximal heart rate (HR) [30], power spectra in specific frequency bands of the heart rate variability (HRV) signal [31], galvanic skin response (GSR) [32], eye-related measures [33], and cortisol levels [34]. However, the aforementioned measures suffer from several practical disadvantages. GSR, HR, and HRV measurements require connections to external sensors that may interfere with task performance [35] and may even be considered obtrusive [36]. These measures also suffer from measurement latency, which is the time gap between the stressful event and the observed response [37,38,39,40].

At this point, it should be noted that many simulators and trainers of psychomotor tasks include some kind of joystick, a computer mouse, or some other handheld human-machine interface used for executing the task. Therefore, monitoring the intensity of a trainee’s hand grip could be a useful method of stress measurement. Wagner et al. [41] examined the feasibility of grip force as a measure of stress in a simulator tracking tasks. Grip force was higher in the stress manipulation presence, showing an equivalent pattern to GSR and self-reported stress. This study provided initial evidence to the ability to distinguish between stressful and non-stressful conditions in physical tracking tasks by measuring the grip force. Botzer et al. [42] recently showed that stress can be detected within 5 seconds through grip force measurement in a simulator. In our previous work [43] we confirmed that grip force is a valid measure of stress by comparing it to heart rate variability indices of stress and by associating both grip force and HRV measures with several intensities of stressful driving events. Additionally, we found that a two-second time window was sufficient for recognizing the stressful events.

In the current research we aim to investigate the usefulness of a stress adaptive psychomotor training system, which utilizes grip force as a measure of stress. Grip force’s covertness and rapid responsiveness are expected to enable this training system to continuously adjust to trainees’ stress and thus to improve training efficiency. Our hypotheses are:

**H1.**  *Learning rate in the stress-adaptive group will be higher than in the control groups.*

**H2.**  *Performance level will be higher in the stress-adaptive group than in the control groups.*

## 2. Materials and Methods

### 2.1. Participants

A total of *n* = 70 undergraduate university students volunteered to participate in this study, in return for credits. Of these 70 volunteers, full data were received from 52 participants only due to missing values caused by instability in data collection. All participants had normal or corrected-to-normal vision. Participants’ age ranged between 22 and 29 years (mean 24.79, S.D. 1.6), while 56% of the participants were female. Of all participants, 46% reported having no previous experience in video or in computer games, 50% reported having some experience, and 4% reported having a lot of experience; 11% of the participants reported being left-handed.

### 2.2. Ethical Committee

The study was conducted in accordance with the Declaration of Helsinki and approved by the Institutional Ethics Committee of Ariel University (protocol code AU-ENG-MW-20201110 10 November 2020). Informed consent was obtained from all subjects involved in the study.

### 2.3. Device

#### 2.3.1. Sensors

A self-developed grip force measurement system was used, similar to the grip force measurement systems that were used and validated by us previously [41,42,43], utilizing two “Adafruit Learning Technologies^®^” force sensitive resistor (FSR) sensors attached to both sides of a symmetrical stationary handhold (to fit operation of both left-handed and right-handed participants), and sampled by an Arduino UNO R3 board. Sampling rate was set to 20 Hz.

**Figure 1:**
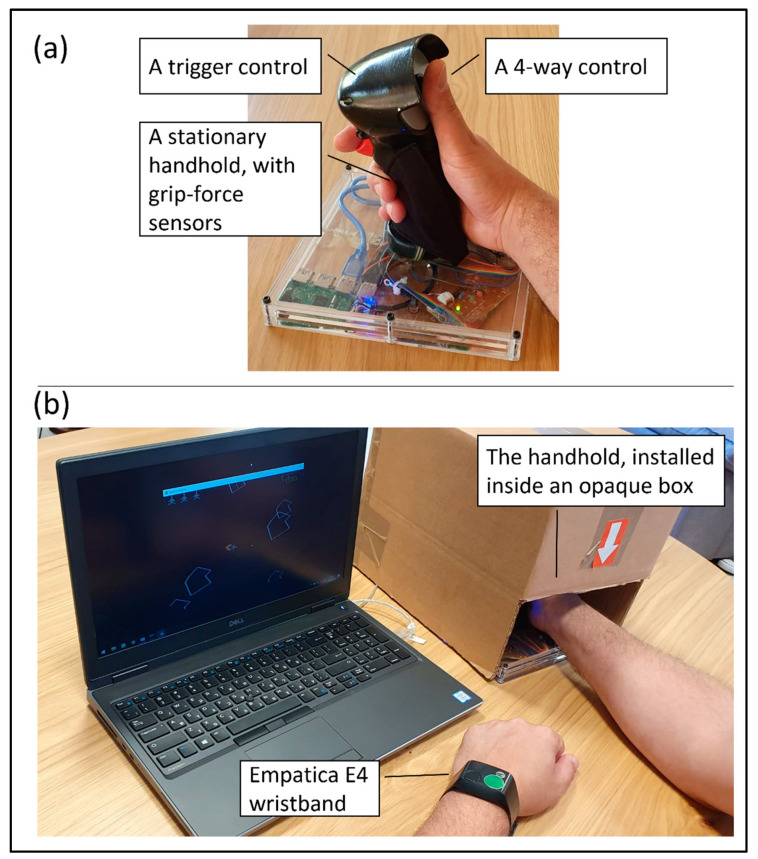
(**a**) The stationary handhold including the grip force sensors and two controls: a 4-way and a trigger; (**b**) general setup, consisting of a laptop with the “Asteroids” game, operated via the controls placed on the handhold, installed inside an opaque box with a hand hole for the right hand and an Empatica E4 wristband on the left hand.

Participants were not told that the grip force was measured. Additionally, the grip force sensors were covered with fabric and concealed from the participants’ view or touch, and the entire handhold was installed inside an opaque box with a hand hole in order to avoid intentional bias as a result of participant’s awareness of the measurement of grip force (see Figure 1). In order to validate stress measurement and calculation according to grip force, we measured participants’ HR using an Empatica E4 wristband placed on the non-dominant hand [44], with a sampling rate of 64 Hz. The measurement of HR is characterized by a latency of tens of seconds in the measurement [29], especially when measured on the wrist. However, the current use was not affected by such delays, since the HR was aggregated for each participant using data covering 30 min. This validation is specified in Appendix A.

#### 2.3.2. Psychomotor Task

**Figure 2:**
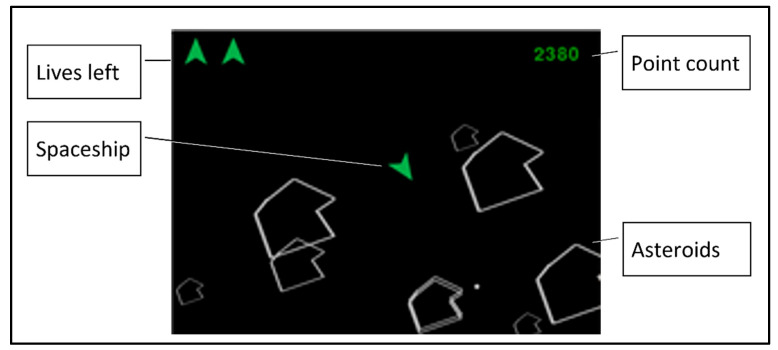
“Asteroids” computer game: typical display.

A two-dimensional Asteroids computer game (downloaded from: https://github.com/aminb/asteroids (accessed on 14 January 2018), see Figure 2) was altered so that it would have 3 possible conditions: constant difficulty level (hereby referred to as “control-constant”), difficulty level that increases with time (hereby referred to as “control-time”), and difficulty level that varies according to the stress level calculated from the grip force (hereby referred to as “stress-adaptive”). Of the valid 52 participants, 41% were in the “stress-adaptive” condition, 31% were in the “control-constant” condition and 28% in the “control-time” condition.

The game was operated by a static operation handle (see Figure 1) with a 4-way control on its top (OTTO’s T4-T) and a trigger control on its frontal surface (OTTO’s U2-025), by the participant’s dominant hand.

In the “Asteroids” game, the operator controls a “spacecraft” that can move forward, turn right or left and shoot (in the direction of its heading). In addition, asteroids appear and move in different bearings and velocities. An encounter of the spacecraft with an asteroid causes the destruction of the spacecraft. To avoid such encounters, the operator can either move the spacecraft or shoot at the asteroids (the latter also entitles the participant to game points). After three encounters between the spacecraft and the asteroids, the game is over. The goal, as the participants were instructed, was to gain the maximal number of points in the game during each experiment session.

Difficulty level was defined as the asteroids’ velocity in a continuous mode (pixel per second, hereby p/s). In the control-constant condition, a constant mid-level velocity (30 p/s) was applied for all asteroids. In the control-time condition, the asteroids’ velocity increased as the game advances (every 5 s the speed increased by 2 p/s). In the stress-adaptive condition, the asteroids’ speed was regulated continuously according to the participant’s current stress level. The stress level was calculated according to the measured grip force on the stationary handhold, using the method described by Wagner et al. [41], adjusted for real-time situations as specified in Equation (1):

(1) $$S=\frac{CGF-SMA}{SMSD}$$

where *S* represents the calculated current stress level of the participant, *CGF* is the current grip force measured value, *SMA* is the simple moving average of grip force (up to 40 min, as specified in Equation (2)), and *SMSD* is the simple moving standard deviation of grip force (up to 40 min), as specified in Equation (3).

(2) $$SMA=\frac{GF_{t}+GF_{t+1}+\ldots +GF_{t+n}}{n}$$

where *SMA* represents the simple moving average of grip force, *GF_t_* is the grip force measured at a time point (*t*), *GF_t_*_+1_ is the grip force measured at the next measurement (after 0.05 s, according to the sampling rate of 20 Hz), and *n* represents the number of grip force samples obtained during the time period of 40 min (i.e., 48,000 samples, according to the sampling rate of 20 Hz).

(3) $$SMSD=\sqrt{\frac{{\left(GF_{t}-SMA\right)}^{2}+{\left(GF_{t+1}-SMA\right)}^{2}+\ldots +{\left(GF_{t+n}-SMA\right)}^{2}}{n}}$$

where *SMSD* represents the simple moving standard deviation of grip force, *SMA* represents the simple moving average of grip force (as specified in Equation (2)), *GF_t_* is the grip force measured at a point time (*t*), *GF_t_*_+1_ is the grip force measured at the next measurement (after 0.05 s, according to the sampling rate of 20 Hz), and *n* represents the number of grip force samples obtained during the time period of 40 min (i.e., 48,000 samples, according to the sampling rate of 20 Hz).

In the stress-adaptive condition, the calculated stress (according to the grip force, as described in Equation (1)) was used to continuously adjust the task difficulty level: when calculated stress level increased above a certain standard deviation (0.5), the task’s difficulty level was lowered (by 2 p/s), and when the calculated stress level dropped below a certain standard deviation (−0.5), the task’s difficulty level was increased (by 2 p/s).

To provide the stress calculation with enough grip force data at the beginning of a game, an initial phase was defined, in which the asteroids’ velocity was constant (6 p/s). This initial 12 seconds’ phase was applied to all other conditions.

Since the game’s average speed (i.e., the task’s difficulty level) varied in the stress-adaptive condition according to each participant’s stress level as measured by grip force, the game’s average speed was controlled for in the control-constant condition. Controlling for the game’s speed was done beforehand to account for alternative interpretations (e.g., that performance differences between the groups were due to differences in game speed). Controlling for the game’s speed was not possible in the control-time condition since in this condition, by its definition, the speed increased over time and could not be adjusted according to any other parameter.

The process of controlling for the game’s speed in the control-constant condition was performed as follows. First, each participant was randomly assigned to one of four batches (each batch consisted of all 3 conditions: stress-adaptive, control-constant, and control-time). Second, in each batch, the first third of the participants were assigned to the stress-adaptive condition. Third, after these participants completed the procedure, their average game speed was calculated. Fourth, the rest of the participants in the batch were randomly assigned to one of the two control conditions (control-constant or control-time). Fifth, the game speed at the control-constant condition was set according to the previously calculated average game speed (as was calculated for the participants in the stress-adaptive condition in the current batch). The same process was conducted for each batch separately.

The performance level was calculated as the natural logarithm of the cumulative hits (i.e., successful shots at the asteroids) minus 10 times the cumulative loss of lives (i.e., asteroids hitting the spacecraft).

### 2.4. Procedure

Upon arriving at the laboratory, each participant was briefed by the experiment supervisor, signed the consent form, and filled in a short questionnaire regarding their relevant personal data (i.e., age, gender, and experience in computer/video games).

The supervisor then described the task and the procedure and provided a demonstration to the participant. All participants completed a two-minute training session, followed by three experimental sessions. Each experimental session included a ten-minute task period and was preceded by a two-minute resting period. All training sessions included the constant-level condition solely. It is emphasized that the participants were not told that grip force was measured, and the handhold with the grip force sensors was hidden from sight by its cover.

### 2.5. Analysis

#### 2.5.1. Required Sample Size

Calculations of the required sample size were done using an R package called “pwr” [45], based on the two Cohen [46] formulas for calculating sample size. The expected effect size was 0.7, according to a meta-analysis of studies concerning training methods [47]. The sample sizes of our experiments were in accordance with the acceptable statistical power for behavioral studies of 0.8 and an acceptable level of significance of 5% [48]. According to this calculation, the required minimum sample size for a single condition is 15, and therefore in the current three-condition setup, the required minimum is 45 participants. Based on this requirement the initial sample size was set at 70 participants to allow for dropout. The final number of participants (*n* = 52) was eventually sufficient.

#### 2.5.2. Analysis Methods

To compare both physiological indices used (i.e., grip force and HR), the mean of each index was first calculated for each participant, and a Pearson r correlation was calculated between both indices in order to examine the linear correlation between these two sets of data. To compare each of the two dependent variables (i.e., “time to criterion”, and “performance at criterion”) between the three condition groups, a linear mixed models (LMM) analysis was employed. In this method, within-subject correlations are modeled using the covariance structure, built on the variance around the outcome measurement and on the correlations between measurements taken from the same participant [49]. In all analyses the significance level was set to less than 5%.

## 3. Results

**Figure 3:**
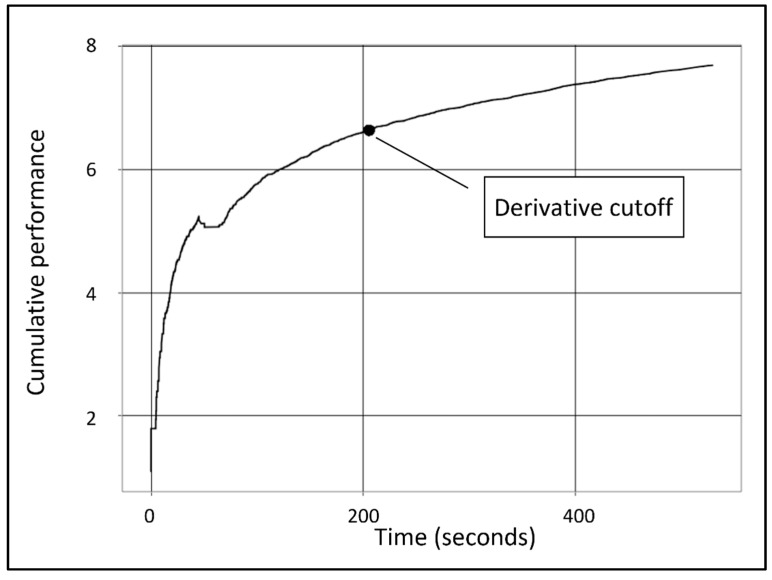
Learning curve for a single participant. X axis represents time (s), Y axis represents performance [Ln (cumulative hits − 10 * cumulative loss of lives)]. Black dot represents the first derivative cutoff point.

To compare the quality of the training, a criterion for the completion of the training is required. First, the learning curve was calculated for each participant (using the R package ‘findCutoff {KneeArrower}’), as demonstrated in Figure 3.

The level of performance was calculated according to the terms explained to the participants at the training phase. As mentioned, the performance level was calculated as the natural logarithm of the cumulative hits (i.e., successful shots at the asteroids) minus 10 times the cumulative loss of lives (i.e., asteroids hitting the spacecraft). A natural logarithm was applied in order to amplify the shape of the learning curve and thus make it easier to notice the cutoff point. The quality of training was determined as two parameters in accordance with the hypotheses: the time required to achieve the training criterion (Hypothesis H1, the X-axis value of the cutoff point) and the level of performance achieved at the training criterion (Hypothesis H2, the Y-axis value of the cutoff point). Calculations of these cutoff points were performed for all 52 participants, in order to get both parameters of training quality (time to criterion and performance at criterion).

**Figure 4:**
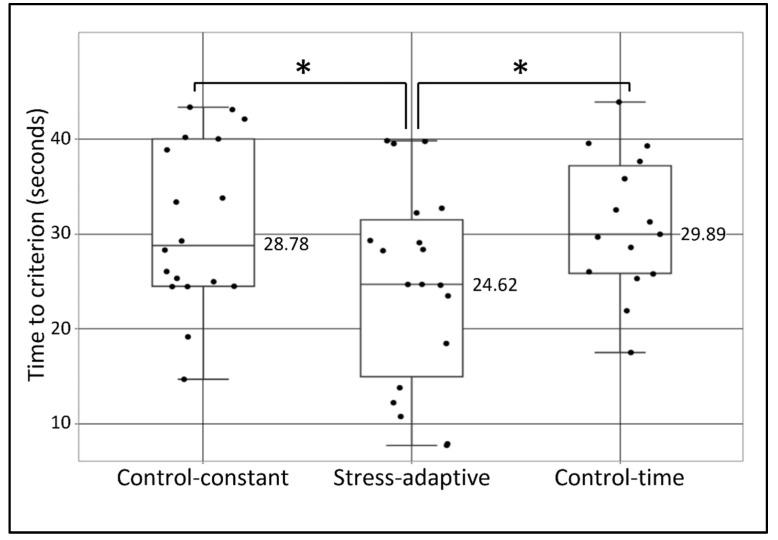
Time to criterion as a function of group. Dots represent the “time to criterion” parameter (from the first derivative cutoff points) for all 52 participants, grouped according to their experimental condition (control-constant, stress-adaptive, and control-time). Boxes represent the inter-quartile range (IQR = Q1 to Q3) of the group, middle horizontal line represents the group’s median, upper line represents the largest value less than upper quartile plus 1.5 times IQR, and the lower line represents the smallest value greater than lower quartile minus 1.5 times IQR. One asterisk represents significance level of *p* ≤ 0.05.

Figure 4 includes a boxplot showing the time to criterion parameter for all participants, divided according to the 3 conditions of the experiment.

From Figure 4, one can notice that the stress-adaptive group showed the shortest required time to achieve the criterion compared with both the control-constant and the control-time. A near-significant effect was found in an LMM analysis for time to criterion F(2,49) = 2.98, *p* = 0.06. The adjusted R^2^ squared was 0.07. A significant main effect of the stress-adaptive condition was found: t = −2.12, *p* = 0.039, which indicates the difference between it and the two control conditions.

**Figure 5:**
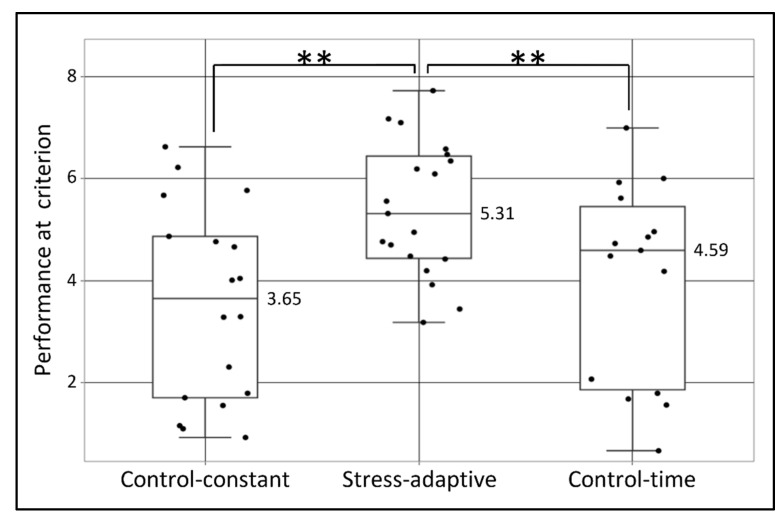
Performance at criterion as a function of group. Dots represent the “performance at criterion” parameter (from the first derivative cutoff points) for all 52 participants, grouped according to their experimental condition (control-constant, stress-adaptive, and control-time). Boxes represent the inter-quartile range (IQR = Q1 to Q3) of the group, middle horizontal line represents the group’s median, upper line represents the largest value less than upper quartile plus 1.5 times IQR, and the lower line represents the smallest value greater than lower quartile minus 1.5 times IQR. Two asterisks represent significance level of *p* ≤ 0.01.

Figure 5 shows a boxplot of the performance level at the criterion parameter for all participants, divided according to 3 conditions of the experiment. From Figure 5 one can easily notice that the stress-adaptive group showed a higher performance level achieved at the criterion, compared with both control-constant and control-time. A significant effect was found in an LMM analysis F(2,49) = 5.78, *p* = 0.006. The adjusted R^2^ was 0.16. A significant main effect of the stress-adaptive condition was found: t = 3.27, *p* = 0.002, which indicates the difference between it and the two control conditions.

No significant effects were found for the background factors (i.e., age, gender, and experience in video games), or for the “time to criterion” or the “performance at criterion” variables.

An LMM analysis was conducted to verify the attempt to compare the speed of the game for the stress-adaptive and control-constant conditions. There was no distinct effect of condition on game speed between these two conditions, a finding that validates our efforts to equalize the conditions of these groups. However, a significant effect was found for the control-time, so that the speed of the game was higher in this condition compared with the other two, F(2,49) = 5.78, *p* = 0.001. Nevertheless, the adjusted R^2^ was 0.006.

## 4. Discussion

The aim of this study was to examine the effect of a stress-adaptive training method, which adjusts the training parameters continuously according to the trainee’s stress level as measured through the grip force on the joystick in a computer game. Stress-adaptive training was compared to two control conditions, either with a constant difficulty or with a time-dependent difficulty of the task. Accordingly, the findings indicate that this innovative training method has certain advantages over other, more conventional training methods in enhancing the quality of training and in shortening the required time of completing the training.

The required time to reach the first criterion (i.e., “derivative cutoff point”, representing the alignment of the learning curve towards its asymptote, in accordance with hypothesis H1) was shorter in the stress-adaptive condition than in both control conditions (constant and time), as evidenced by the finding of a significant main effect of the stress-adaptive condition in relation to the two control conditions: t = −2.12, *p* = 0.039. This finding confirms the first hypothesis. Additionally, the level of performance that participants achieved at the criterion was higher in the stress-adaptive training condition compared to both control conditions (constant and time), according to the significant main effect of the stress-adaptive condition that was found: t = 3.27, *p* = 0.002, which confirms hypothesis H2. It can be concluded, based on these findings, that the stress-adaptive training method is an efficient way of training, capable of improving the final level of performance while shortening the required period of training.

In order to investigate a possible alternative interpretation of these results (i.e., in the stress-adaptive condition participants controlled the speed of the game and thus had an easier task to master), the speed of the game in the control-constant condition was fitted to the average speed of the stress-adaptive condition, so there was no difference in terms of task difficulty between these conditions. Nonetheless, the speed of the control-time condition was significantly higher than the speed of the other two conditions, F(2,49) = 5.78, *p* = 0.001. Therefore, one can argue that the higher performance at the criterion in the stress-adaptive condition, compared with the control-time condition, may be a result of the higher speed of the latter, which means a tougher task. Still, the higher speed at the control-time condition was unavoidable since the very definition of this condition was an increase in speed every given period of time. If there had been a smaller increase in game speed for the control-time condition, it is possible that performance at the criterion would have been higher than the actual one. Future studies may further investigate this possibility. Nevertheless, it is emphasized that since the adjusted R^2^ of this effect was 0.006, it can be concluded that this possible effect was too small to impact the main findings. Other alternative explanations were also considered, such as the effect of age, gender, and previous experience in video/computer games. However, these effects were not significant, which reduces the probability that these factors influenced the results.

According to our findings, this new training technique has the potential of elevating performance at the end of a training period for psychomotor tasks similar to the one presented in this research. Future tools may further analyze the levels of stress during the task execution, allowing reflection on the course of training and provide insights for improving training sessions. An alternative approach of elaborating on this training method is to utilize machine learning (ML) abilities, which are capable of forming a complex model based on multiple sources of information [50]. Such advanced tools may consider not only the online information regarding the level of stress of the trainee, but also the level of performance at any given moment, and therefore adjust the training features accordingly, to achieve optimal training. Another potential future endeavor in SAT is its application in high-fidelity training environments to further explore this training method efficiency and its relevance for practical use, although the additional benefit of such an approach is still unclear [51].

These findings provide the opportunity of improving the efficiency of psychomotor training in various fields such as aviation, driving, and robotic surgery [52]. A particularly interesting potential in our findings regards the final level of performance achieved in the SAT condition. Should this finding be reproduced in future studies, its possible implication is that SAT may aid in training higher level experts possessing abilities superior to those of today’s reasonable experts. Finally, these results affirm that stress measurement using grip force has practical implications, along with its advantages over other physiological stress measures, namely being nonintrusive and suitable for continuously measuring stress. It should also be noted that the described apparatus consists of inexpensive components, which are rather easy to assemble and employ. This economical training tool may aid in improving training outcomes, thus bringing a greater value to the costs invested.

It should be noted that there are certain limitations to this study. First, since the relationship of performance and stress is a well-studied one [53,54], it can be assumed that had the apparatus taken the performance factor into account during the execution, the training results might have been improved and the required time period to reach the criterion might have been even shorter. Second, stress was calculated for the adaptivity purpose according to the grip force solely. Although the results indicate that this measure correlated with heart rate and was sufficient to improve training, a more comprehensive approach for stress measurement that utilizes several stress indices may aid in the determination of a more accurate level of stress, thus leading to better training results.

## 5. Conclusions

In the current study, an innovative stress adaptive training method led to better training results, with shorter training duration required, compared with more traditional training methods. The main effects were on the required training duration for achieving the criterion (t = −2.12, *p* = 0.039) in accordance with the first hypothesis as well as on the level of performance reached at the criterion (t = 3.27, *p* = 0.002) in accordance with the second hypothesis. This stress-adaptive training method has the potential of improving training and experts’ performance levels in various psychomotor domains, thereby providing a practical use for grip force as a measure of stress.

## Figures and Tables

**Figure A1:**
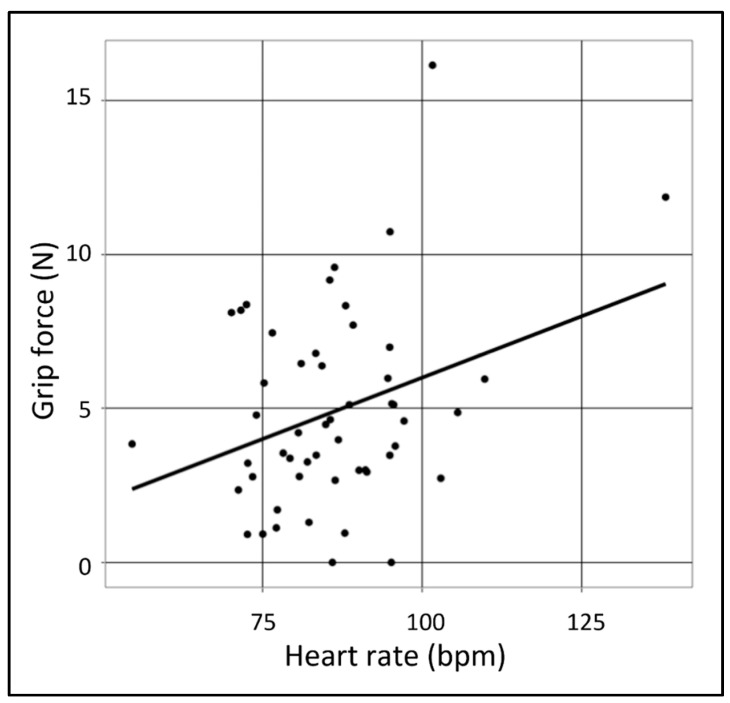
Grip force as a function of heart rate. The plot illustrates the significant correlation where X-axis values represent the mean heart rate (bpm), and the Y-axis values represent mean grip force (N). Dots represent individual observations (each participant’s values). Line represents smoothed conditional means using lm smoothing.

## Data Availability

The data presented in this study are available on request from the corresponding author. The data are not publicly available due to the privacy of the research participants.

## Appendix A

For validation purposes of grip force as a stress measure for the current task, grip force was correlated with heart rate, an established measure of stress. A Pearson correlation was found significant r (52) = 2.77, *p* = 0.007, as demonstrated in Figure A1.

## References

[1] Darwin C. (1987). Charles Darwin’s Natural Selection: Being the Second Part of His Big Species Book Written from 1856 to 1858.

[2] Georgopoulos A.P. (2000). Neural aspects of cognitive motor control. Curr. Opin. Neurobiol..

[3] Bruzzone A.G., Massei M. (2017). Simulation-based military training. Guide to Simulation-Based Disciplines.

[4] Papagiannakis G., Trahanias P., Kenanidis E., Tsiridis E. (2018). Psychomotor surgical training in virtual reality. The Adult Hip-Master Case Series and Techniques.

[5] Knoke B., Gorldt C., Thoben K.D. (2019). Literature Review on Training Simulators. Manufacturing Processes.

[6] Jentsch F., Curtis M. (2017). Simulation in Aviation Training.

[7] Bradley P. (2006). The history of simulation in medical education and possible future directions. Med. Educ..

[8] Landsberg C.R., Van Buskirk W.L., Astwood R.S., Jr., Mercado A.D., Aakre A.J. (2010). Adaptive Training Considerations for Use in Simulation-Based Systems.

[9] Redei A. (2019). Applications and Evaluation of a Motion Flight Simulator. Ph.D. Dissertation.

[10] Yoon S., Park T., Lee J.H., Kim J.A. Study on Transfer Effectiveness and Appropriate Training Hours in Airplanes Simulators. Proceedings of the ITEC.

[11] Dębska M., Polechoński J., Mynarski A., Polechoński P. (2019). Enjoyment and Intensity of Physical Activity in Immersive Virtual Reality Performed on Innovative Training Devices in Compliance with Recommendations for Health. Int. J. Environ. Res. Public Health.

[12] Schaffernak H., Moesl B., Vorraber W., Koglbauer I.V. (2020). Potential Augmented Reality Application Areas for Pilot Education: An Exploratory Study. Educ. Sci..

[13] Stadnicka D., Litwin P., Antonelli D. (2019). Human factor in intelligent manufacturing systems-knowledge acquisition and motivation. Procedia CIR.

[14] Zahabi M., Razak A.M.A. (2020). Adaptive virtual reality-based training: A systematic literature review and framework. Virtual Real.

[15] Plott B.M., McDermott P.L., Archer S., Carolan T.F., Hutchins S., Fisher A., Orvis K.A. (2014). Understanding the Impact of Training on Performance.

[16] Couvillion K.F., Bass A.D., Fairbrother J.T. (2020). Increased cognitive load during acquisition of a continuous task eliminates the learning effects of self-controlled knowledge of results. J. Sports Sci..

[17] McKendrick R., Harwood A. (2019). Cognitive Workload and Workload Transitions Elicit Curvilinear Hemodynamics During Spatial Working Memory. Front. Hum. Neurosci..

[18] Mark J., Thomas N., Kraft A., Casebeer W.D., Ziegler M., Ayaz H. (2017). Neurofeedback for Personalized Adaptive Training. International Conference on Applied Human Factors and Ergonomics.

[19] Smith N.D., Gunnink E.D., Schnell T., Reuter C., Moss J.D. Workload-Adaptive Training Scenarios for Synthetic Training Environments. Proceedings of the Sixth Annual GIFT Users Symposium, US Army Research Laboratory.

[20] Schroeder B.L., Fraulini N.W., Marraffino M.D., Van Buskirk W.L., Johnson C.I. Individual Differences in Adaptive Training: Distress, Workload, and Coping with Changes in Difficulty. Proceedings of the Human Factors and Ergonomics Society Annual Meeting Sage CA.

[21] McEwen B.S. (2000). Definitions and concepts of stress. Encyclopedia of Stress, 3.

[22] Staal M.A. (2004). Stress, Cognition, and Human Performance: A Literature Review and Conceptual Framework.

[23] Evans G.W., Johnson D. (2000). Stress and open-office noise. J Appl. Psychol..

[24] Tichon J.G., Wallis G.M. (2010). Stress training and simulator complexity: Why sometimes more is less. Behav. Inf. Technol..

[25] Cohen S., Weinstein N. (1981). Nonauditory effects of noise on behavior and health. JSI.

[26] Theologus G.C., Wheaton G.R., Fleishman E.A. (1974). Effects of intermittent, moderate intensity noise stress on human performance. J. Appl. Psychol..

[27] Warm J.S., Dember W.N., Hancock P.A., Parasuraman R., Mouloua M. (1996). Vigilance and workload in automated systems. Automation and Human Performance.

[28] Yerkes R.M., Dodson J.D. (1908). The relation of strength of stimulus to rapidity of habit formation. J. Comp. Neurol..

[29] Contrada R.J., Baum A. (2011). The Handbook of Stress Science: Biology, Psychology, and Health.

[30] Kudielka B.M., Schommer N.C., Hellhammer D.H., Kirschbaum C. (2004). Acute HPA axis responses, heart rate, and mood changes to psychosocial stress (TSST) in humans at different times of day. Psychoneuroendocrinology.

[31] Giannakakis G., Grigoriadis D., Giannakaki K., Simantiraki O., Roniotis A., Tsiknakis M. (2019). Review on psychological stress detection using biosignals. IEEE Trans. Affect. Comput..

[32] Al-Fudail M., Mellar H. (2008). Investigating teacher stress when using technology. Comput. Educ..

[33] Matthews G., Reinerman-Jones L.E., Barber D.J., Abich IV J. (2015). The psychometrics of mental workload: Multiple measures are sensitive but divergent. Hum. Factors.

[34] Yamaguchi M., Wakasugi J., Sakakima J. Evaluation of driver stress using biomarker in motor-vehicle driving simulator. Proceedings of the International Conference of the IEEE Engineering in Medicine and Biology Society.

[35] Healey J., Picard R.W. (2005). Detecting stress during real-world driving tasks using physiological sensors. IEEE Trans. Intell. Transp. Syst..

[36] Dinges D.F., Rider R.L., Dorrian J., McGlinchey E.L., Rogers N.L., Cizman Z., Metaxas D.N. (2005). Optical computer recognition of facial expressions associated with stress induced by performance demands. ASEM.

[37] Bruun A. It’s not complicated: A study of non-specialists analyzing GSR sensor data to detect UX related events. Proceedings of the 10th Nordic Conference on Human-Computer Interaction.

[38] Kirschbaum C., Hellhammer D.H. (1994). Salivary cortisol in psychoneuroendocrine research: Recent developments and applications. Psychoneuroendocrinology.

[39] Kucera P., Goldenberg Z., Kurca E. (2004). Sympathetic skin response: Review of the method and its clinical use. Bratisl Lek Listy..

[40] Nickel P., Nachreiner F. (2003). Sensitivity and diagnosticity of the 0.1-Hz component of heart rate variability as an indicator of mental workload. Int. J. Hum. Factors Ergon..

[41] Wagner M., Sahar Y., Elbaum T., Botzer A., Berliner E. (2015). Grip Force as a Measure of Stress in Aviation. Int. J. Aviat. Psychol..

[42] Botzer A., Sahar Y., Wagner M., Elbaum T. (2021). Analyzing individuals’ grip force over short intervals in a joystick-controlled task with and without a stress manipulation. Behav. Inf. Technol..

[43] Sahar Y., Elbaum T., Wagner M., Musicant O., Hirsh T., Shoval S. (2021). Grip Force on Steering Wheel as a Measure of Stress. Front. Psychol..

[44] Ollander S., Godin C., Campagne A., Charbonnier S. A comparison of wearable and stationary sensors for stress detection. Proceedings of the 2016 IEEE International Conference on systems, man, and Cybernetics (SMC).

[45] Champely S., Ekstrom C., Dalgaard P., Gill J., Weibelzahl S., Anandkumar A., Ford C., Volcic R., De Rosario H. (2020). pwr: Basic Functions for Power Analysis. https://cran.r-project.org/web/packages/pwr/.

[46] Cohen J. (1988). Statistical Power Analysis for the Behavioral Sciences.

[47] Martinelli A., Grüll J., Baum C. (2022). Attention and interpretation cognitive bias change: A systematic review and meta-analysis of bias modification paradigms. Behav. Res. Ther..

[48] Fritz M.S., MacKinnon D.P. (2007). Required sample size to detect the mediated effect. Psychol. Sci..

[49] Peat J., Barton B. (2014). Medical Statistics: A Guide to SPSS, Data Analysis and Critical Appraisal.

[50] Leite D., Martins A., Rativa D., De Oliveira J.F., Maciel A. (2022). An Automated Machine Learning Approach for Real-Time Fault Detection and Diagnosis. Sensors.

[51] van Weelden E., Alimardani M., Wiltshire T.J., Louwerse M.M. (2022). Aviation and neurophysiology: A systematic review. Appl. Ergon..

[52] Nagyné Elek R., Haidegger T. (2021). Non-technical skill assessment and mental load evaluation in robot-assisted minimally invasive surgery. Sensors.

[53] Anderson C.R. (1976). Coping behaviors as intervening mechanisms in the inverted-U stress-performance relationship. J. Appl. Psychol..

[54] Hancock P.A., Warn J.S. (1989). A dynamic model of stress and sustained attention. Hum. Factors.

